# National Preferred Interpersonal Distance Curbs the Spread of COVID-19: A Cross-Country Analysis

**DOI:** 10.1017/dmp.2020.295

**Published:** 2020-08-12

**Authors:** Yunus Gokmen, Ufuk Turen, Haluk Erdem, İsmail Tokmak

**Affiliations:** Turkish Military Academy, Ankara, Turkey; Baskent University, Ankara, Turkey

**Keywords:** corona virus, COVID-19, interpersonal distance, pandemics, social distancing

## Abstract

**Objectives:**

National interpersonal distance preference is considered a cultural characteristic. Interpersonal distance is critical for the spread dynamics of coronavirus disease 2019 (COVID-19). COVID-19’s spread trend shows various characteristics in different countries. We believe that 1 of the factors influencing this variation could be national interpersonal distance preference.

**Methods:**

We used regression analysis based on data of national interpersonal distance preferences (social, personal, and intimate) presented by Sorokowska et al. and COVID-19 rate of spread data for 40 different countries that were calculated using Our World in Data’s data.

**Results:**

National interpersonal distance preferences, with its 3 dimensions, significantly influence the rate of spread of COVID-19 in countries.

**Conclusion:**

Understanding the relation between national interpersonal distance preference and spread of COVID-19 might be very useful information in decision-making processes of individuals, societies, and governments to develop culturally well-suited counter-pandemic policies, strategies, and procedures during the COVID-19 pandemic or any epidemic or pandemic threats in the future, instead of standard fit-to-all strategies.

The disease caused by the novel coronavirus, which is believed to have started in Wuhan, China, in December 2019 and distinguished as a “pandemic” by the World Health Organization (WHO) in March 2020, is called novel coronavirus disease 2019 (COVID-19). Once the novel coronavirus propagated exponentially throughout the world and, in a couple of weeks, turned into one of the worst pandemics in history, the health systems of most nations were caught unprepared and suffered under the pressure of the exponential growth in the numbers of infected patients, especially the ones needing intensive care and respiratory support.^1^

Science is needed in this race run against time to halt the disease spread and its impact on the lives of tens of thousands of people without questioning social class or nationality. Although there are still many unknowns regarding COVID-19 and ways to fight it, we now know some of its transmission dynamics between humans and other indirect routes. Despite the general assumptions on viral spread by respiratory droplets and safe interpersonal distance of 2 meters, novel coronavirus can be transferred through fomites and aerosols, meaning safe interpersonal distance should be greater.^2,3^ As a basic and classic strategy to contain the virus, most countries applied quarantine, isolation, or curfew policies to stop people from gathering and socializing, to mitigate the transmission of virus from one person to another.

Meanwhile, we noticed an interesting difference between nations in the rate of spread, despite sharing often the same social values and understanding of hygiene, presence of similar national health systems, and significant investment in science and technology. There might be many factors influencing the spread and impact of an infectious disease, such as the time at the first case enters the country, the robustness of national prevention and mitigation measures and policies applied, the strength of the health system, different testing strategies, active contact-tracing procedures, and other means and capabilities.^4^ However, nations’ social habits, such as social contacts and mixing patterns, are also considered as credible factors in the process.^5^ It is also revealed that nations have different preferred interpersonal distancing habits (eg., Hall and Sorokowska et al.).^6,7^ Because the rapid spread of human-to-human transmitted COVID-19 is critically sensitive to the physical distance between individuals,^8^ we believe that the social habits of a nation relative to preferred interpersonal distance could be effective in the spreading process of COVID-19. Based on this idea, we explored the literature and could not find any research addressing this relationship. To fill the gap in the literature, we decided to explore the association between national interpersonal distance preferences and rate of COVID-19 spread in different societies.

Understanding the relation between preferred physical distance between individuals in a society and rate of spread of COVID-19 might be very useful information to be used in decision-making processes of individuals, societies, and governments to develop their counter-pandemic politics, strategies, and procedures during this COVID-19 pandemic or any epidemic or pandemic threads in the future. By doing so, well-suited plans and strategies can be tailored based on the cultural preferences at all levels instead of standard manufactured fit-to-all strategies.

## BACKGROUND AND HYPOTHESES

### Interpersonal Distance

Proxemic Theory’s interpersonal distance concept,^6^ which is seen as an essential feature of bilateral relations between individuals^9^ is considered an important form of nonverbal communication.^10^ Behaviors related to interpersonal distance are often related to the protection of certain proximities that people perceive as private.^11^

The basic dimensions of these invisible borders differ according to cultural, social, personality, and environmental variables, and these borders ensure that the appropriate distance is maintained between individuals.^12,13^ This physical distance is seen as a tendency to approach or avoid social stimuli^14^ that often make people feel uncomfortable and threatened when that space is invaded.^15^ Interpersonal distance is sensitive not only to personal attitude toward another but also to gender,^16^ age,^17^ status and power,^18^ and culture.^19^

The concept of “social distance” is based on Simmel’s^20^ theory of the stranger in the early 20th century. As a follower of Simmel, Bogardus^21^ proposes that individuals focus on their feelings toward each other, and defined social distance as a function of the degree of mutual sympathetic understanding, and establishes a scale of “social distance” beginning with the level of “someone to marry” and ending with “someone to exile from the country.” Apart from this view, Hall’s^6^ Proxemic Theory uses the term “interpersonal distance” and posits that it is a physical distance kept as personal breathing space, like an invisible bubble, that surrounds an individual representing an imaginary barrier to regulate intimacy with others by controlling the proximity of visual, tactile, auditory, and olfactory stimulation. This theory is based on the senses and mutual bodily stimulation effects between individuals, and focuses on the individual and his or her close approximations to others. The Proxemic Theory categorizes interpersonal distance in 4 intervals: (1) *Intimate distance (0 in. - 18 in.);* all senses are active and there is love, family, or close friend relationships in between. (2) *Personal distance (1.5 ft - 4 ft);* is the minimum comfortable distance between nontouching individuals, and this zone constitutes a small protective space. (3) *Social distance (4 ft - 12 ft);* is considered as the area where individuals do not care about each other and do not threaten each other. The distance of individuals at workplaces without disturbing each other can be given as an example. (4) *Public distance (12 ft and beyond);* is the distance at which one can take either evasive or defensive action if physically threatened. Formal situations such as conference and speech can be given as examples. To prevent possible confusion in terms, the term “social distance,” which is used by Bogardus, is also used by Hall to describe the third interval in his scale. It is believed that there is no intellectual influence between these 2 theoretical approaches, although they both explore almost the same aspect of social life.^22^

In addition, Hall^6^ categorizes cultures in 2 sets: contact and noncontact. Contact cultures are more inclined to touch each other, preferring shorted interpersonal distance while noncontact cultures show antipodal attitudes. He uses geographical division and suggests that contact culture dominates Southern European, Latin American, and Arabic and noncontact culture prevails in North American, Northern European, and Asian populations.

Little^23^ compares perception of personal space of Swedish, Scottish, and American people with that of Greek and Italian people and finds that perception of personal space of Greek and Italian people is narrower. Watson^24^ in his research conducted on foreign students living in the United States, separates the students into 2 groups, similar to Hall,^6^ according to countries with high contact culture (Arabs, Latin Americans, South East Europeans) and those with low contact culture (North Americans, North Europeans, Pakistanis, and Asians) and investigates their perception of personal space. He reveals that personal spaces of students from countries with contact culture are narrower than personal spaces of those from countries without contact culture.

Remland et al.^25^ affirm that English, French, and Dutch people prefer greater personal space than Italian and Greek people. Beaulieu^26^ sorts different cultures in terms of largest personal space size preference order: Anglo-Saxons come first, Asians second, and Caucasians third, and Mediterranean people and Latinos prefer the shortest distance. Tiechuan^27^ claims that interpersonal distance preference for Spanish or Arabic people is much closer than for British people, and interpersonal distance for people in Western culture is greater than Eastern culture. Ke and Lian^28^ report that interpersonal distance preferences differ between Chinese and American people. While Americans consider only spouses and children in their intimate space, Chinese people accept many more people in theirs. People waiting in crowded queues keep their personal spaces in American Society, whereas these spaces can be as close as physical contact in Chinese Society in similar circumstances. The impact of climate on interpersonal distance preferences is also examined. The individuals dwelling in hot climates are found to be friendlier and warmer than those living in cold climates (eg, Wei et al., Ven de Viliert, Ke and Lian, Sorokowska et al.).^7,28-30^

### Interpersonal Distance and Contagion

From a medical perspective as a preventive measure, the interpersonal distance concept is frequently used as social distancing and being strongly advised to slow down the spread of contagion causing epidemics or pandemics (eg Ahmed et al., Fong et al., WHO, ECDC).^31-34^ Studies focusing on the spread dynamic of other epidemics and pandemics in history also suggest the spread rate of contagious virus can be significantly restrained by practice and policies that emphasize social distancing (eg, Caley et al.).^35^

During the COVID-19 pandemic, social distancing beyond the transmission range of virus is considered a key factor to stop virus spread from an infected individual to a healthy one and to flatten the curve of contagion spread to keep the total number of cases at any given time within the health systems’ means and capabilities.^36,37^ The failure of society to practice social distance measures effectively often led to much more draconian and extremely costly governmental measures, such as quarantine, curfew, and lockdown to inhibit the speed of contagion spread.^38,39^ Some governments have delayed to take social distancing-related measures or gave inconsistent and confusing messages to people, making them perplexed,^40^ and some societies and groups simply resist the advised or instructed policies (eg, McMurtry and Zampano, Perrone).^41,42^

During our literature review, we find that there is one study exploring the association between interpersonal distance habits and the mortality caused by tuberculosis. This study used cross-country data and reported that greater national interpersonal distance preferences diminished the tuberculosis fatality rate.^43^ However, we cannot locate any study examining the relation between national interpersonal distance preference and spread rate of an infection during a pandemic.

We believe that this relation is critical for understand the social aspects of COVID-19 spread dynamics and useful to develop tailored preventive and mitigation plans for different societies and also notable to fill the gap in the literature. Hence, we theorize a greater COVID-19 spread rate in countries where people prefer shorter interpersonal distances compared with countries where they prefer greater distances, and we developed the hypotheses below:

H_1_: Society’s preferred interpersonal distance (total) influences COVID-19 spread rate.
H_2_: Society’s preferred social distance influences COVID-19 spread rate.
H_3_: Society’s preferred personal distance influences COVID-19 spread rate.
H_4_: Society’s preferred intimate distance influences COVID-19 spread rate.

### Methods and Data

For national interpersonal distances, we used national interpersonal distances preference as function of 3 variables, namely social distance (SD), personal distance (PD), and intimate distance (ID) of countries around world, which was measured by Sorokowska et al.^7^ with a pioneering and comprehensive study, including 8943 participants from 42 different countries. For estimating interpersonal distance, participants were asked to fill a questionnaire including 3 graphical questions related to their preferred interpersonal distance. However, although the data set of national interpersonal distances data is not shown, we estimated sensitively using pixel/millimetric coordinate system approach (Jasc Paint Shop 8.0) based on the scaled graphic presented in the study. Although there are 4 intervals of interpersonal distance in Hall’s model,^6^ we think the first 3 of them are the relevant approximations for viral transmission. Public distance, which is considered 12 ft and beyond is excluded from our research scope. This interval is not measured by Sorokowska et al.^7^ as well.

To measure the increase in rate of spread of COVID-19, we used a simple exponential growth function (Equation 1), which is used for forecasting of the spread of an early phase of pandemic as stated by Anderson and May,^44^ Chowell et al.,^45^ Wallinga and Lipsitch,^46^ and Viboud et al.^47^
(1)
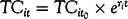



*TC*_*it*_ : Total COVID-19 cases of i^th^ country related to t^th^ day (April 7^th^ 2020),

*TC*_*it0*_ : Total COVID-19 cases of i^th^ country related to the date of reaching the 100^th^ cumulative case,

*r*_*i*_ : The growth rate related to i^th^ country.

To compute the growth rate (r) for each country, data on COVID-19 total cases (covering the period between the date of reaching the 100th cumulative case and April 7, 2020) are collected from Our World in Data (OWD) formal website,^48^ which sorts out daily COVID-19 data from the WHO situation reports. Hong Kong and Uganda are excluded from analysis, due to lack of COVID-19 total cases data. The data set is presented in Appendix A.

### Data Analysis

We examined the impacts of national interpersonal distances (social, personal, and intimate distance) on the growth rate of total cases of COVID-19 (GRTC) totally and separately. To see the total effect, the average interpersonal distance of each nation is computed by using the geometric mean formula. Additionally, because the dependent and independent variables in the models have different measurement units, a simple logarithmic regression model is proposed, as stated in Equation 2-5. Regression models are powerful tools to estimate and/or predict the dependent variable’s (population) mean by using 1 or more independent variables.^49^
(2)
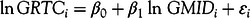

(3)
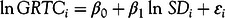

(4)
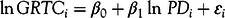

(5)
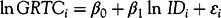



*GRTC*_*i*_ *: The natural logarithm of Growth Rate of Total Cases of COVID-19 of i*^*th*^ *country*;

*GMID*_*i*_ *: The natural logarithm of geometric mean of interpersonal distance values of i*^*th*^ *country*;

*SD*_*i*_ *: The natural logarithm of Social Distance value of i*^*th*^ *country*;

*PD*_*i*_ *: The natural logarithm of Personal Distance value of i*^*th*^ *country*;

*ID*_*i*_ *: The natural logarithm of Intimate Distance value of i*^*th*^ *country*;

ε_*i*_ *: The error (residual) term in the regression model.*

## RESULTS

To test the hypotheses, simple regression analysis is conducted for each model. The summary of the regression models and coefficients is illustrated in Table 1. We see that each national interpersonal distance value (GMID, SD, PD, and ID) has a significant and negative impact on GRTC. The results of regression models show that all coefficients in the models are significant at the α = 0.05 level, and the directions of the associations are in line with our hypotheses.

**TABLE 1:** Summary of Regression Analyses^a,b,c^

Mod./Hypt. ID	R^2^	Adjusted R^2^	SE	F	*P*-Value	Coeff.	Unstandardized Coefficients		Standardized Coefficients	t	*P*-Value	Hypotheses Results
							β_i_	SE	β_i_			
1	0.198	0.176	0.0516	9.354	0.004^d^	(Constant)	2.789	1.577		1.768	0.085	Accepted
						lnGMID	-1.113	0.364	-0.444	-3.058	0.004^d^	
2	0.193	0.171	0.0518	9.066	0.005^d^	(Constant)	3.063	1.693		1.809	0.078	Accepted
						lnSD	-1.100	0.365	-0.439	-3.011	0.005^d^	
3	0.183	0.161	0.0521	8.503	0.006^d^	(Constant)	2.819	1.665		1.694	0.099	Accepted
						lnPD	-1.113	0.382	-0.428	-2.916	0.006^d^	
4	0.111	0.088	0.0543	4.768	0.035^d^	(Constant)	0.279	1.060		0.264	0.794	Accepted
						lnID	-0.578	0.265	-0.334	-2.184	0.035^d^	

a Dependent variable: lnGRTC.

b In order to test normality assumption, one-sample Kolmogorov-Smirnov normality test (The null hypothesis (H_0_): The data of the variable is distributed normality) is performed, as the test values of data of all variables are greater than α = 0.05 level, the null hypothesis (H_0_) is accepted.

c For testing heteroscedasticity assumption, White homoscedasticity test (H_0_: There is no heteroscedasticity in the model) is employed, since all test values higher than α = 0.05 level, it can be deduced that there is no heteroscedasticity in the models.

d The regression model and coefficients are significant at α = 0.05 level.

## DISCUSSION AND CONCLUSIONS

In epidemics or pandemics, the spread rate of an infection is considered a major characteristic producing destruction in societies. Containing the spread is simply an effort to keep the number of severe cases needing intensive care within the capacity of health systems, gain time to increase health systems capacity, and develop drugs and vaccines.^50^ Today, distance kept among individuals to break the contagion chain is a classic and most-effective method for buying the time needed.

Our analyses show that there is a significant and negative (opposite) relation between national interpersonal distance preferences and COVID-19 spread rate. Based on Hall’s Proxemic Theory,^6^ we separately used 3 sub-concepts, namely social, intimate, and personal distance preferences of nations to examine the impact of each on COVİD-19 spread rate and determined that each increment of increase in distance for each variable leads to a decrease in COVID-19 spread rate. Findings support our hypotheses and are in line with the only previously published study examining the relation between national interpersonal distance preference and tuberculosis fatality rate.^43^ In addition, the findings are supported by the medical prevention and mitigation measures and advice mentioned above (eg, ECDC, 2020),^34^ and the behavior of COVID-19 spread making Italy and Spain the epicenter in Europe, among many other nations.^41^ Thus, we can say that national interpersonal distance as 1 variable, and increases in each of its 3 dimensions significantly decrease the rate of spread of COVID-19 in a country. In other words, nations with greater interpersonal distance preference are less vulnerable to COVID-19 than the ones with shorter interpersonal distance preference.

We think that this study is important because it supports the social dimension of pandemics. Interpersonal distance preferences of nations and their social and cultural properties are sustained but slowly change over time. Being cognizant of these cultural behavioral patterns’ impact on medical phenomena is critical. Apart from technical and medical precautions and measures, this social aspect having an impact on viral transmission dynamics can be key for decision-makers, authorities, and ordinary people. If governments use this information during their decision-making processes related to pandemics, such as COVID-19 and upcoming epidemic or pandemic threats, they can develop more realistic, tailored, and effective plans reflecting the habits and preferences of their citizens. In this regard, we can suggest that, in societies with shorter interpersonal distance preferences, governments should take much more draconian and early preventive measures and remind people about their habits and invite them to practice greater interpersonal distance. If there is no information about the facts, correction efforts are often inappropriate and useless. Governments unaware of the interpersonal distance preference of their pertinent society often use standard measures widely accepted in the international community but not well tailored for their particular community.

We also think our findings are valuable because they promote the idea of a social or interpersonal distancing strategy in fighting COVID-19 through providing cross-country evidence supporting its effective functionality.

This study is the first study examining the relation between national interpersonal distance preferences and the spread rate of an infectious disease. In this respect, it is valuable because it fills an important gap in the literature. Furthermore, we believe that the relations between the social behavior of people and pandemic-related matters should be explored further to understand the variance among different societies. Only by understanding social science can we appreciate the contribution of human behavior and culture in this phenomenon and learn lessons for all.

Our study has some limitations. We used secondary data in our analyses. The data on rate of spread is derived from OWD,^48^ and national interpersonal distance preference data are from Sorokowska et al.^7^ So, we assume that the data presented by these sources are true and accurate. COVID-19 rate of spread is based on the data collected during a limited time interval, by April 7, 2020. Because the pandemic is still spreading globally, COVID-19 rates can vary over time. Our data source for national interpersonal distance preference, Sorokowska et al.,^7^ addresses 42 countries; therefore, our analyses are limited to those societies. These limitations should be considered before any generalizations.

Pandemics are also likely to impact interpersonal distance preference (eg, Faulkner et al.),^51^ and this relation is also valuable to understand the impact of the COVID-19 pandemic on societies. Efforts to measure national interpersonal distance preferences after the COVID-19 pandemic could be very useful to see the variation between before and after, if any, and recalibrate the international database for the concept.

## APPENDIX A

**Table:** Data Set of Dependent and Independent Variables^a^

ID	Country	Dependent Variable^b^	Independent Variables (cm)^b^			
		Growth Rate of Total Cases (GRTC)	Geometric Mean of Interpersonal Distance (GMID)	Social Distance (SD)	Personal Distance (PD)	Intimate Distance (ID)
1	Argentina	0.1338	57.45	77.33	59.33	41.33
2	Austria	0.1597	67.47	88.00	68.00	51.33
3	Brazil	0.1917	75.38	101.33	77.33	54.67
4	Bulgaria	0.0871	62.25	82.67	65.33	44.67
5	Canada	0.1884	85.80	102.67	84.67	72.67
6	China	0.0743	82.14	114.67	83.33	58.00
7	Colombia	0.1370	84.37	116.00	85.33	60.67
8	Croatia	0.1297	90.36	108.67	89.33	76.00
9	Czech Republic	0.1434	79.83	110.00	80.67	57.33
10	Estonia	0.0944	91.23	117.33	93.33	69.33
11	Germany	0.1788	65.94	96.00	70.00	42.67
12	Ghana	0.0647	78.51	106.67	82.00	55.33
13	Greece	0.0992	66.77	92.00	69.33	46.67
14	Hungary	0.1151	104.69	129.33	107.33	82.67
15	India	0.1621	82.39	110.00	86.67	58.67
16	Indonesia	0.1330	84.96	110.00	85.33	65.33
17	Iran	0.1482	82.12	112.00	83.33	59.33
18	Italy	0.1571	64.70	93.33	68.00	42.67
19	Kazakhstan	0.1433	67.69	94.67	67.33	48.67
20	Kenya	0.0724	82.18	110.00	88.00	57.33
21	Malaysia	0.1200	73.28	110.00	76.67	46.67
22	Mexico	0.1514	81.26	99.33	82.67	65.33
23	Nigeria	0.0746	80.36	102.67	80.67	62.67
24	Norway	0.1228	65.39	104.00	72.00	37.33
25	Pakistan	0.1377	84.43	118.00	90.00	56.67
26	Peru	0.1470	61.63	80.67	64.00	45.33
27	Poland	0.1562	68.25	96.67	66.67	49.33
28	Portugal	0.1861	73.63	110.00	76.67	47.33
29	Romania	0.1492	84.09	134.67	92.00	48.00
30	Russia	0.1914	67.57	89.33	74.00	46.67
31	Saudi Arabia	0.1332	107.76	125.33	104.00	96.00
32	Serbia	0.1505	65.32	92.67	67.33	44.67
33	Slovakia	0.0804	63.72	88.67	67.33	43.33
34	South Korea	0.0894	83.30	105.33	84.00	65.33
35	Spain	0.1966	72.55	90.00	74.00	57.33
36	Switzerland	0.1449	91.03	110.00	92.67	74.00
37	The United Kingdom	0.1850	76.86	99.33	80.67	56.67
38	The United States	0.2273	68.30	95.33	68.67	48.67
39	Turkey	0.2532	87.37	122.67	92.67	58.67
40	Ukraine	0.1890	61.14	86.00	65.33	40.67

a Hong Kong and Uganda are excluded from analysis, due to lack of COVID-19 total cases data. The decimal parts of independent variables are rounded to 2 digits.

b In order to ensure normality and homoscedasticity assumptions, transformation methods (logarithm and simple smoothing) are conducted before regression analysis.
